# MDMF: Predicting miRNA–Disease Association Based on Matrix Factorization with Disease Similarity Constraint

**DOI:** 10.3390/jpm12060885

**Published:** 2022-05-27

**Authors:** Jihwan Ha

**Affiliations:** Major of Big Data Convergence, Division of Data Information Science, Pukyoung National University, Busan 48513, Korea; jhha@pknu.ac.kr; Tel.: +82-51-629-4614

**Keywords:** microRNA, disease, matrix factorization, miRNA–disease association

## Abstract

MicroRNAs (miRNAs) have drawn enormous attention owing to their significant roles in various biological processes, as well as in the pathogenesis of human diseases. Therefore, predicting miRNA–disease associations is a pivotal task for the early diagnosis and better understanding of disease pathogenesis. To date, numerous computational frameworks have been proposed to identify potential miRNA–disease associations without escalating the costs and time required for clinical experiments. In this regard, I propose a novel computational framework (MDMF) for identifying potential miRNA–disease associations using matrix factorization with a disease similarity constraint. To evaluate the performance of MDMF, I calculated the area under the ROC curve (AUCs) in the framework of global and local leave-one-out cross-validation (LOOCV). In conclusion, MDMF achieved reliable AUC values of 0.9147 and 0.8905 for global and local LOOCV, respectively, which was a significant improvement upon the previous methods. Additionally, case studies were conducted on two major human cancers (breast cancer and lung cancer) to validate the effectiveness of MDMF. Comprehensive experimental results demonstrate that MDMF not only discovers miRNA–disease associations efficiently but also deciphers the underlying roles of miRNAs in the pathogenesis of diseases at a system level.

## 1. Introduction

MicroRNAs (miRNAs) are a type of non-coding RNA consisting of 19–22 nucleotides. miRNAs have been reported to be involved in the regulation of gene expression at the post-transcriptional level. They bind to the 3′ untranslated regions (UTRs) of target mRNAs through base paring [1,2,3]. Since the first two miRNAs were discovered, Caenorhabditis elegans lin-4 and let-7, and numerous more miRNAs have been discovered thanks to the high-throughput techniques [4,5]. Furthermore, increasing evidence indicates that miRNAs play crucial roles in various biological processes. For example, miRNAs are found to be involving in aging [6], apoptosis [7], cell development [8] differentiation [9], and proliferation [10]. As such, abnormalities and dysfunction of miRNAs may be involved in various disease incidents, including cancers, cardiovascular diseases, and nervous system disorders [11]. For example, miR-21 has been found to play a significant role in regulating the expression of MAP2K3, a tumor repressor gene related to hepatocellular carcinoma cell proliferation [12]. In addition, studies have shown that mir-31 and mir335 are involved in suppressing breast cancer [13,14,15]. Therefore, it is necessary to determine the role of miRNAs as biomarkers, which could not only improve the understanding of disease pathogenesis but also contribute to the treatment and detection of complex human diseases. In this regard, efforts have focused on identifying the relationship between miRNAs and diseases through biological experiments such as microarray profiling and qRT-PCR. However, considering the cost and complexity of biological experiments, computational approaches for studying disease-related miRNAs may be a good alternative for reducing the time and money required for clinical methods.

Most previous computational approaches have predicted miRNA–disease associations based on the biological hypothesis that functionally related miRNAs tend to be associated with phenotypically similar diseases [16]. The most intuitive way of reflecting this assumption is to construct a miRNA similarity network, where nodes represent the miRNAs and edges represent functional similarities among the miRNAs. Potential disease-related miRNAs can be identified by observing their neighbors in the miRNA similarity network. In this regard, several computational methods have been proposed to efficiently identify disease-related miRNAs. Xuan et al. proposed a computational model called HDMP for predicting miRNA–disease associations by considering the network using k-nearest neighbors in the network [16]. HDMP identified potential disease-related miRNAs based on the assumption that miRNAs in the same cluster or family tend to be associated with phenotypically similar diseases. However, HDMP cannot be applied to miRNAs with no disease associations. Jiang et al. developed a hypergeometric distribution-based prediction model by employing a miRNA functional similarity network, a disease phenotype similarity network, and a human miRNA–disease association network [17]. However, this model limits further improvement by considering only the local information in the network. Mørk et al. presented a prediction model of miRPD that utilizes miRNA–disease associations and disease–protein associations. This model has the prediction power of predicting both disease-related miRNAs and proteins. [18]. In miRPD, proteins are used to link the associations between miRNAs and diseases. However, their high dependency on protein information limits the application of miRNAs with no protein links. Chen et al. developed the computational model called random walk with restart for miRNA–disease association (RWRMDA) [19]. RWRMDA is based on the assumption that applying global information in a network better captures miRNA–disease associations than using only local information. To infer novel miRNA–disease associations, a random walk with a restart algorithm was implemented in a pre-constructed miRNA functional similarity network (MFSN), until the probability of each node became stable. However, this model still fails to predict miRNAs with no disease association, which limits further improvement. Chen et al. developed another prediction framework of WBSMDA [20]. WBSMDA integrated various heterogeneous biological datasets such as known Gaussian interaction profile kernel similarity, disease semantic similarity network, miRNA functional similarity network, and known miRNA–disease associations. Compared to previous models, WBSMDA could effectively work on new miRNAs with no disease associations and diseases with no miRNA associations, which improved prediction accuracy. Chen et al. developed a prediction model called the HGIMDA [21]. By integrating various similarity values, such as miRNA functional similarity, disease semantic similarity, Gaussian interaction profile kernel, HGIMDA identified miRNA–disease associations by searching three-length paths in the heterogeneous network. Shi et al. developed a computational framework for disease-related miRNA prioritization by implementing a random walk on a protein–protein interaction (PPI) network [22]. In this study, miRNA target genes and causal genes of diseases were mapped onto the PPI network to investigate miRNA–disease associations. Ha et al. developed a network-based model for identifying disease-related miRNAs based on the assumption that functionally similar miRNAs tend to share a large proportion of common environmental factors (EFs) [23]. EFs are known to be important for miRNA regulation. However, this model can improve performance by considering the chemical compounds among EFs. In summary, the aforementioned similarity-based methods are highly dependent on disease-related miRNAs, which limits their further improvement.

Owing to recent advancements, machine learning is being widely used in various areas of research [24,25,26,27]. To elucidate the role of miRNAs in tumorigenesis and disease pathogenesis, considerable efforts have been made to reveal miRNA–disease associations based on machine learning models. The following miRNA–disease association prediction method can be categorized into machine leaning-based models. Ha et al. used matrix factorization to identify novel disease-related miRNAs [28]. This model effectively enhanced performance by applying miRNA expression as a weight for the matrix factorization objective function. Chen et al. proposed a computational model called a restricted Boltzmann machine for inferring potential miRNA–disease associations, for multiple types of miRNA–disease association predictions (RLSMDA) [29]. Semi-supervised learning was performed to predict novel disease-related miRNAs without the use of negative samples. However, choosing the appropriate parameters for the classifiers from two different spaces remains a problem. Chen et al. also proposed the prediction model called the RBMMMDA [30], which is a two-layer undirected graphical model consisting of visible layers and hidden units. RBMMMDA is not only capable of predicting disease-related miRNAs, but also determining the corresponding types. Li et al. proposed a matrix completion-based model for miRNA–disease association prediction called MDMDA [31], which uses a singular value thresholding (SVT) algorithm based on a binary adjacency matrix. Xio et al. presented a computational model called GRNMF. In this study, the authors measured the interaction profiles of diseases and miRNAs using a weighted gene network [32]. Chen et al. further developed a novel computational model to infer potential miRNA–disease associations, called IMCMDA [33]. By measuring comprehensive similarities among miRNAs and diseases, IMCMDA performed satisfactorily in detecting disease-related miRNAs. Chen et al. also proposed a model called MDHGI, which identifies potential miRNA–disease associations using a matrix decomposition algorithm based on the Gaussian interaction profile kernel, disease semantic similarity, and miRNA functional similarity [34]. Chen et al. developed a model called RKNNMDA that uses support vector machine by exploring the k-nearest neighbors of diseases and miRNAs to prioritize disease-related miRNAs based on weighted voting [35].

Predicting miRNA–disease associations can be regarded as the type of problem suited for recommender systems, where the goal is to infer the most plausible rating scores that a user might assign to a certain item. Among the various machine-learning algorithms, matrix factorization has achieved immense success in recommender systems. Thus, various models have been developed to transform the task of prediction potential miRNA–disease associations into a recommender task [36,37]. The key idea of matrix factorization is to find two non-negative matrices, called latent spaces, whose product approximates the observed value in the original matrix. In other words, they map miRNAs and diseases into a shared latent space to represent a vector of latent features. After the optimization process, the inner product of each latent space can be used to identify the relationship between miRNAs and diseases.

Various matrix factorization-based models have been developed to enhance prediction accuracy by injecting additional information, a process called implicit feedback. Because of high-throughput techniques, large amounts of biological data are now readily available, which helps decipher the underlying roles of miRNAs in pathological and physiological activities. Therefore, it is necessary to take considerations of using additional biological data, a notion that led to the design of the matrix factorization architecture in this study.

Here, I propose an effective and feasible computational framework to predict miRNA–disease associations via matrix factorization with disease similarity constraint (MDMF). MDMF is a pioneering method that leverages known miRNA–disease associations, miRNA expression values, and disease semantic similarity, and it identifies potential miRNA–disease associations by satisfying the constraint that the cosine similarity of the disease latent space should be close to the value of the disease semantic matrix. As a result, MDMF achieved AUCs of 0.9147 and 0.8905 in the framework of global and local leave-one-out cross-validation (LOOCV), respectively, which is superior to previous methods. Furthermore, a literature analysis of the top 50 candidate miRNAs related to breast and lung cancers clearly demonstrated the effectiveness of MDMF.

## 2. Materials and Methods

**Figure 1:**
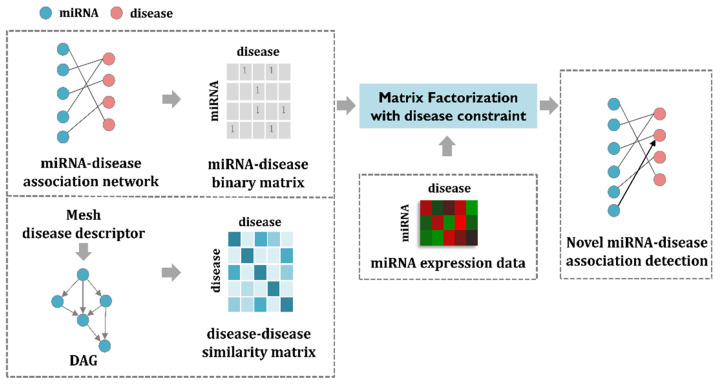
The workflow of MDMF. First, a miRNA–disease association matrix is constructed based on the known miRNA–disease association network. In a similar way, a disease-disease similarity matrix is created through the mesh descriptor. Second, by combining miRNA expression value as a weight for the objective function, the matrix factorization model is efficiently learned. Finally, through the MDMF, each miRNA is given a score for association with a disease, which is used as a basis for making actual predictions.

In this section, I first enumerate the datasets used for the MDMF and then formulate the matrix factorization model to address the task of predicting miRNA–disease associations. The MDMF can be divided into three steps. First, a miRNA–disease association matrix is constructed based on the known miRNA–disease association network. In a similar way, a disease–disease similarity matrix is created through the mesh descriptor. Second, by combining miRNA expression value as a weight for the objective function, the matrix factorization model is efficiently learned. Finally, through the MDMF, each miRNA is given a score for association with a disease, which is used as a basis for making actual predictions. The main goal of MDMF is to predict miRNA–disease associations. In other words, MDMF determines whether each miRNA is associated with a disease or not. Therefore, predicting the relationship between miRNA and disease can be considered as binary classification. The overall workflow of MDMF is shown in Figure 1.

### 2.1. Human miRNA–Disease Association Data

With the discovery of numerous miRNAs through accumulated evidence, various databases have been developed to store information regarding these miRNAs. Hence, the author obtained human miRNA–disease associations from public databases (HMDD v3.2, dbDEMC, and miR2Disease). HMDD v2.0 contains 5430 known miRNA-disease associations for 495 miRNAs and 383 diseases that are based on verified experimental results [38]. dbDEMC v2.0 contains information on miRNA–disease associations regarding on 2224 miRNAs and 36 diseases [39]. miR2disease contains information on 3723 miRNA–disease associations for 349 miRNAs and 136 diseases [40]. The author removed duplicate entities and unified disease names by utilizing the MeSH disease terms. Each dataset contains information indicating the relationship between miRNA and disease. It can be considered as binary information representing disease and miRNA relationships. By integrating each data set, it was used as original miRNA–disease association matrix for matrix factorization. The original miRNA–disease association matrix can be defined as:

(1) $$y_{ui}=\{\begin{array}{c} 1,\;\;\;\mathrm{miRNA}i\mathrm{and}\;\mathrm{disease}i\mathrm{is}\;\mathrm{related}\\ 0,\;\;\;\;\;\;\;\;\;\;\mathrm{otherwise} \end{array}$$

### 2.2. miRNA Expression Data

Despite the success of matrix factorization in machine learning, it suffers from an inherent limitation that lowers prediction accuracy owing to the lack of observations (i.e., known miRNA–disease associations), that lowers prediction accuracy. Owing to high-throughput techniques, vast amounts of omics data are now publicly available, which provide clues for predicting potential links between miRNAs and diseases. To this end, using miRNA expression values could be a great alternative for uncovering the hidden functions of miRNAs involved in disease pathogenesis. A value of 0 in the original matrix does not necessarily indicate that miRNA m is not related to disease d; it is possible that this relationship has not yet been revealed. Therefore, the author assigned miRNA expression values for entries that did not have known miRNA–disease associations. MiRNA expression value consists of numerical information and it was normalized through min–max normalization. When a specific condition is given (disease), a high miRNA expression value means that the condition and miRNA are correlated. The miRNA expression data were obtained from The Cancer Genome Atlas (TCGA), which provides comprehensive proteomic, epigenomic, genomic, and transcriptomic data [41].

### 2.3. Disease Semantic Similarity

In this study, the author used a hierarchical directed acyclic graph (DAG) to calculate similarities between diseases. Generally, a directed acyclic graph (DAG) can be utilized to model the likelihood of pairwise relationships between two nodes. By introducing DAG, which captures the semantics behind disease similarities, I was able to calculate precise similarities among diseases, which is paramount for enhancing the performance in the identification of disease-related miRNAs. Disease *D* can be expressed as DAG(*D*) = (*D*, *T(D)*, E(*D*)). *T(D)* stands for the ancestor nodes of node *D*, including node *D* itself, and E(*D*) denotes its direct edges from general terms (parent nodes) to more specific terms (child nodes) [42]. The author downloaded the Disease Mesh descriptor from the National Library of Medicine (http://www.nlm.nih.gov) to construct the hierarchical directed acyclic graph [43], where the disease semantic similarity *D* can be expressed as follows:

(2) $$DV(D)=\sum_{t\in T(D)}D_{D}(d)$$

(3) $$\{\begin{array}{c} D_{D}(d)=1\\ D_{D}(d)=\max\{\Delta_{*}D_{D}(d^{\prime })|d^{\prime }\in children\;of\;d\}\;if\;d\;\neq \;D \end{array}$$

where Δ is the semantic contribution factor. The author assigned a higher disease semantic similarity value if a disease pair shared a larger portion of DAGs. The disease semantic similarity between *d(i)* and *d(j)* can be calculated as follows:

(4) $$SS_{1}\left(d\left(i\right),d\left(j\right)\right)=\frac{\sum_{t\in T\left(i\right)\cap^{\;}T\left(j\right)}\left(D_{i}\left(t\right)+D_{j}\left(t\right)\right)}{DV\left(i\right)+DV\left(j\right)}$$

### 2.4. Gaussian Interaction Profile Kernel Disease Similarity

Gaussian interaction profile (GIP) kernel similarities for diseases are calculated based on the biological hypothesis that phenotypically similar diseases tend to associate with functionally similar miRNAs [37]. The binary vector *IP(d(i))* represents the interaction profiles of disease *d(i)*, which can be obtained from known associations between disease *d(i)* and each miRNA. The GIP similarity between *d(i)* and *d(j)* is defined as follows:

(5) $$GS(d(i),d(j))=\exp(-r_{d}||IP(d(i))-IP(d(j))|{|}^{2})$$

Here, *r_d_* denotes the hyperparameter for the bandwidth of the kernel that can be measured by normalizing *r^’^_d_*. *r^’^_d_* represents the average number of related miRNAs per disease.

(6) $$r_{d}=\frac{r_{d}^{\prime }}{\frac{1}{n_{d}}\sum_{i=1}^{n_{d}}||IP(d(i)|{|}^{2}}$$

### 2.5. Integrated Disease Similarity

Owing to the reason that disease semantic similarities do not cover all the similarities between the diseases, the author combined the disease semantic similarity SS and Gaussian interaction kernel similarity GS for obtaining comprehensive disease similarities. The integrated similarity between *d(i)* and *d(j)* is calculated as follows:

(7) $$\begin{array}{l} S_{d}(d(i),d(j))\\ =\{\begin{array}{c} SS(d(i),d(j)),\;if\;d(i)\;and\;d(j)\;has\;semantic\;similarity\\ GS(d(i),d(j),\;\;\;\;\;\;\;\;\;\;\;\;otherwise \end{array} \end{array}$$

### 2.6. EMDMF

**Figure 2:**
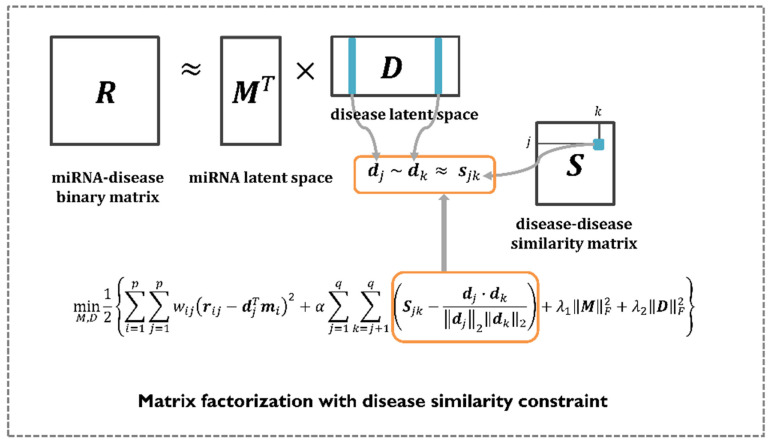
The workflow of MDMF. MDMF learns the two latent spaces whose products are close to the observed entries in the original matrix, whereas the cosine similarity of the disease latent space is close to the value of the integrated disease similarity matrix.

Matrix factorization has shown great performance in recommender systems, where its goal is to predict the most plausible rating scores that a user might give to certain items. Therefore, numerous computational models use matrix factorization to perform various research tasks, including the identification of disease-related miRNAs. The aim is to find two non-negative matrices, called latent spaces, whose product approximates the observed value in the original matrix. In this study, the author mathematically formulated the problem of predicting miRNA–disease associations by employing matrix factorization with a disease similarity constraint. Unlike previous methods, MDMF leverages integrated disease similarities to represent a more precise disease latent space. In summary, the goal of MDMF is to learn the two latent spaces whose products are close to the observed entries in the original matrix, whereas the cosine similarity of the disease latent space is close to the value of the integrated disease similarity matrix. This learning process is illustrated in Figure 2. Equation (8) illustrates the design of a matrix factorization objective function with a disease similarity constraint. All the notations are listed in Table 1.

(8) $$\underset{M,D}{\min}\frac{1}{2}\{\sum_{i=1}^{p}\sum_{j=1}^{q}w_{ij}{\left(r_{ij}-d_{j}^{T}m_{i}\right)}^{2}+\alpha \sum_{j=1}^{q}\sum_{k=j+1}^{q}\left(S_{jk}-\frac{d_{j}\cdot d_{k}}{{\left\|d_{j}\right\|}_{2}{\left\|d_{k}\right\|}_{2}}\right)\;+\lambda_{1}{\left\|M\right\|}_{F}^{2}+\lambda_{2}{\left\|D\right\|}_{F}^{2}\}$$

I computed the gradient of each latent vector and trained it using the gradient descent algorithm. The gradients are calculated as follows:

(9) $$\frac{\partial L}{\partial m_{i}}=\sum_{j=1}^{q}w_{ij}(d_{j}^{T}m_{i}-r_{ij})d_{j}+\lambda_{1}m_{i}$$

(10) $$\frac{\partial L}{\partial d_{j}}=\sum_{i=1}^{p}w_{ij}(d_{j}^{T}m_{i}-r_{ij})m_{i}+\lambda_{2}d_{j}+\alpha \sum_{k=j+1}^{q}\{\left(S_{jk}-\frac{d_{j}\cdot d_{k}}{{\left\|d_{j}\right\|}_{2}{\left\|d_{k}\right\|}_{2}}\right)\;\left(-\frac{1}{{\left\|d_{k}\right\|}_{2}}\right)\left(\frac{d_{k}}{{\left\|d_{j}\right\|}_{2}}-\frac{d_{j}\cdot d_{k}}{{\left\|d_{j}\right\|}_{2}^{3}}d_{j}\right)\}$$

Each miRNA disease latent space was updated in the opposite direction of the gradient with a magnitude proportional to the learning rate. This procedure can be expressed as follows:

(11) $$i=1\;to\;p:\;m_{i}\leftarrow m_{i}+\eta \left\{\sum_{j=1}^{q}w_{ij}(d_{j}^{T}m_{i}-r_{ij})d_{j}+\lambda_{1}m_{i}\right\}$$

(12) $$j=1\;to\;q:\;d_{j}\leftarrow d_{j}+\eta \{\sum_{i=1}^{q}w_{ij}(r_{ij}-d_{j}^{T}m_{i})m_{i}-\lambda_{2}d_{j}\}\;+\alpha \sum_{k=j+1}^{q}\left\{\left(S_{jk}-\frac{d_{j}\cdot d_{k}}{{\left\|d_{j}\right\|}_{2}{\left\|d_{k}\right\|}_{2}}\right)\left(-\frac{1}{{\left\|d_{k}\right\|}_{2}}\right)\left(\frac{d_{k}}{{\left\|d_{j}\right\|}_{2}}-\frac{d_{j}\cdot d_{k}}{{\left\|d_{j}\right\|}_{2}^{3}}d_{j}\right)\right\}$$

Through Equation (12), the author aims to explicitly inject the integrated disease similarities into the disease latent space to obtain a more precise disease latent vector representation. Using the gradient descent algorithm, I could learn latent spaces (i.e., miRNA, disease) and identify potential miRNA–disease associations by taking the dot product of each latent space. All the notations are listed in Table 1.

## 3. Results

### 3.1. Evaluation Metric

To demonstrate the performance of the MDMF, the author implemented leave-one-out cross-validation (LOOCV) as an evaluation metric. In general, LOOCV can be considered as a special type of n-fold cross validation, in which each known miRNA–disease association is left out as a test sample, while the other samples are used as training samples [44]. Two types of LOOCV exist: global LOOCV and local LOOCV. Global LOOCV considers all diseases at once, whereas local LOOCV considers only one specific disease at a time. The main difference between global LOOCV and local LOOCV depends on whether all diseases were tested at the same time or not. By plotting the true positive rate (TPR, sensitivity) versus the false positive rate (FPR, 1-specificity), the author drew receiver operating characteristic (ROC) curves at different thresholds. In the ROC graph, the *X*-axis denotes the true positive rate (TPR) and the *Y*-axis represents the false positive rate (FPR). In general, the area under the ROC curve (AUC) is widely used to evaluate the performance of the model; AUC = 1 indicates perfect prediction performance and AUC = 0.5 denotes random selection [45]. In addition, several evaluation metrics including area under the precision-recall curve (AUPR), F1-measure (F1), accuracy (ACC), and Matthews correlation coefficient (MCC) were adopted to demonstrate the performance. PR curves are widely used in the field of machine learning, where unbalanced datasets are more often observed. Because of the imbalanced data sets, the PR curve is becoming a useful alternative, which can highlight the performance differences lost in the ROC curve. Accuracy refers to the number of correctly identified disease-related miRNAs among the selected miRNAs. Both the Matthews correlation coefficient (MCC) and F1 enable to establish the consistency of experimental results between both metrics and the proportion that change.

### 3.2. Performance Comparison with Previous Methods

**Figure 3:**
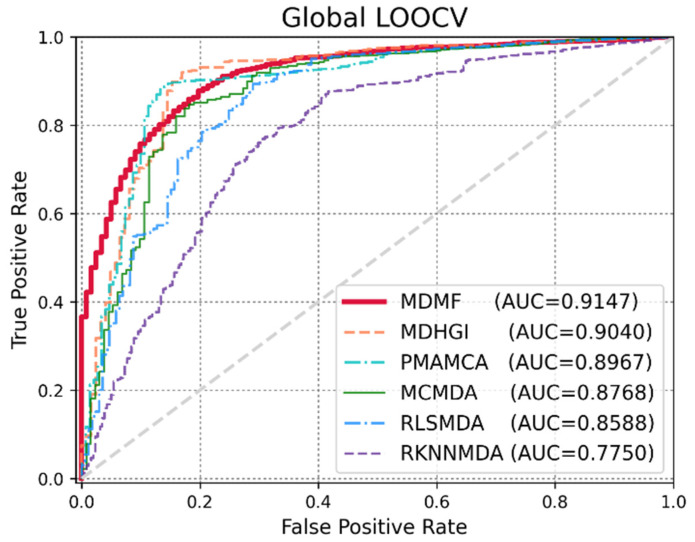
Performance comparisons between MDMF and five existing miRNA–disease association prediction models (MDHGI, PMAMCA, MCMDA, RLSMDA, and RKNNMDA) in terms of AUCs based on global LOOCV.

**Figure 4:**
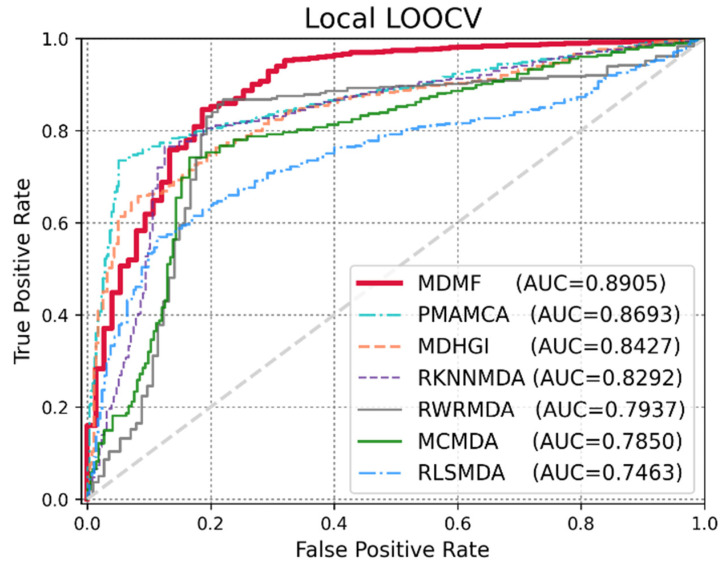
Performance comparisons between MDMF and six existing miRNA–disease association prediction models (PMAMCA, MDHGI, RKNNMDA, RWRMDA, MCMDA, and RLSMDA) in terms of AUCs based on local LOOCV.

Global LOOCV was used to demonstrate the performance enhancement by MDMF in predicting known miRNA–disease associations over existing state-of-the-art techniques. Comparative experiments were performed based on the source code provided in each paper. In other cases, the author directly implemented the code and implemented the experiments. As shown in Figure 3, MDMF, MDHGI [34], PMAMCA [28], MCMDA [31], RLSMDA [29], and RKNNMDA [35] obtained AUCs of 0.9147, 0.9040, 0.8967, 0.8768, 0.8588, and 0.7750, respectively, within the framework of global LOOCV. Next, I employed another evaluation metric local LOOCV to evaluate the robustness of MDMF in identifying known miRNA–disease associations. As shown in Figure 4, MDMF, PMAMCA [28], MDHGI [34], RKNNMDA [35], RWRMDA [23], MCMDA [31], and RLSMDA [29] achieved AUCs of 0.8905, 0.8693, 0.8427, 0.8292, 0.7937, 0.7850, and 0.7463, respectively, within the framework of local LOOCV. Furthermore, I performed additional experiments by adopting several other evaluation metrics, including area under the precision-recall curve (AUPR), Matthews correlation coefficient (MCC), F1-measure (F1), and accuracy (ACC). In conclusion, the experimental results (Table 2 and Table 3) of the various evaluation metrics clearly demonstrate the superior performance of MDMF compared to the previous state-of-the-art approaches.

### 3.3. Effect of Disease Similarity Constraint

**Figure 5:**
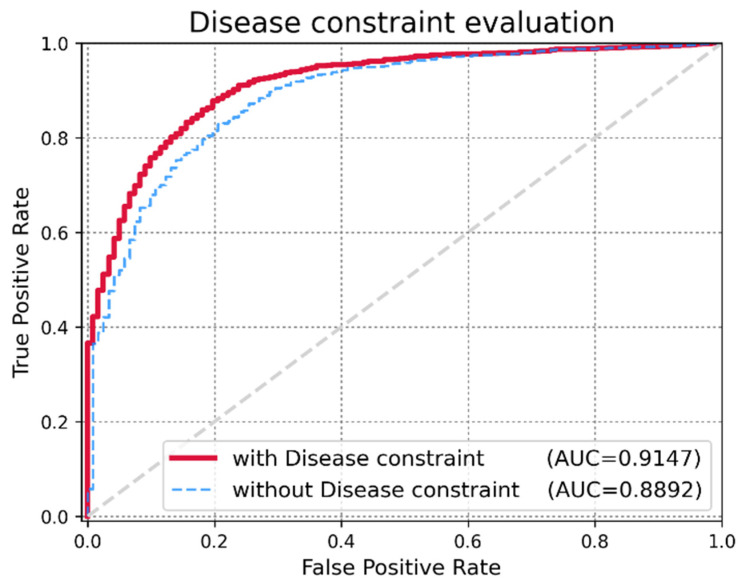
Performance evaluation of MDMF with and without the disease constraint. Application of the disease constraint increased AUC score.

An important criterion for evaluating the expandability of MDMF is whether the model achieves high performance while injecting a disease similarity constraint. The author assigned α as a hyper-parameter that controls the trade-off between the original matrix factorization and the disease similarity constraint. In extreme cases, if I employ a very small value of α, I only mine miRNA–disease associations and miRNA expression values for matrix factorization. However, if I employ a very large value of α, the integrated disease similarity information dominates the learning process. To demonstrate the effectiveness of injecting the disease similarity constraint, I evaluated changes in the AUCs by varying the value of α. As shown in Figure 5, a higher α yielded better performance. However, beyond a certain point (α = 0.7), the performance degraded. Therefore, the author fixed the optimal hyper-parameter value of α to 0.7 for our experiments. The experimental results for the various parameters are listed in Table 4.

### 3.4. Case Studies (Breast Cancer and Lung Cancer)

To confirm the robustness and effectiveness of MDMF in predicting miRNA–disease associations, the author conducted case studies on two major human cancers in context of the miRNA research: breast cancer and lung cancer.

Cancer is associated with abnormal cell growth, which can spread to nearby tissues. Among the various human cancers, breast cancer is a malignant neoplasm and is the most common cancer type among women. Therefore, I implemented a case study of breast cancer to demonstrate the performance of MDMF and prioritized the top 50 candidates based on the relatedness scores assigned by MDMF. As shown in Table 5, all 50 candidates were verified as true breast cancer-related miRNAs based on the answer set data. The author confirmed that all top 50 candidates were involved in breast cancer-related processes.

Lung cancer is the leading cause of cancer-related deaths in both men and women [46]. The dominant factor leading to lung cancer is exposure to cigarette smoke through active or passive smoking. Epidemiological research on lung cancer has demonstrated that miRNAs also play an important role in its pathogenesis. Therefore, I further carried out MDMF to predict potential lung cancer-related miRNAs. As shown in Table 6, the author verified that all top 50 miRNAs were related to lung cancer.

### 3.5. Survival Analysis

**Figure 6:**
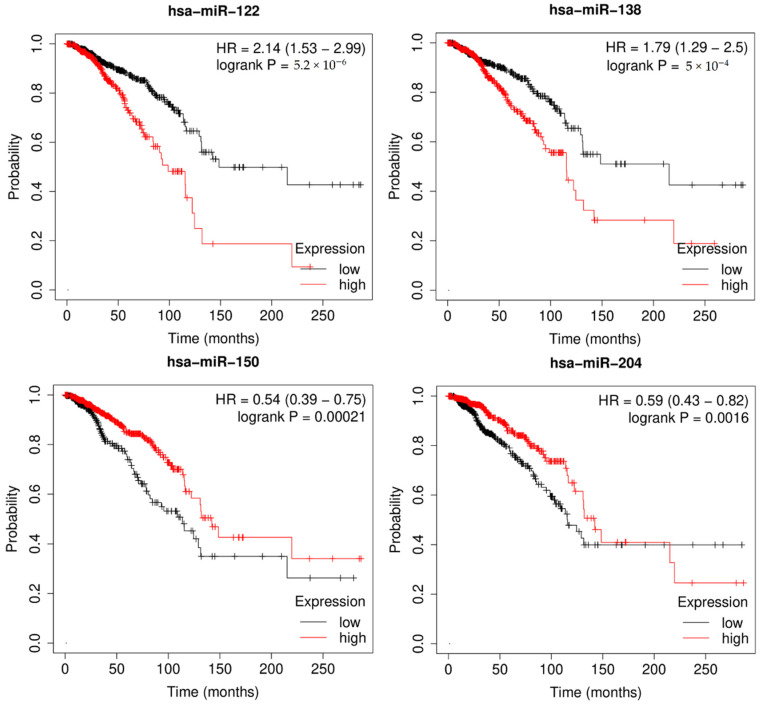
Kaplan–Meier plots of hsa-miR-520e, hsa-miR-593, hsa-miR-381, and hsa-miR-500a, for survival of breast cancer patients. Survival analysis demonstrated effectiveness of MDMF in discovering potential biomarkers.

Survival analysis, also called event analysis, corresponds to the statistics for investigating the time it takes for some event of interest to occur. It is widely used in biomedical research, where the objective is to observe the time to death of patients. Therefore, I implemented Kaplan-Meier survival analysis to verify the effect of miRNAs on overall patient survival using the miRpower-Kaplan-Meier plotter web-tool [47]. Based on the top 50 candidates, miRNAs with a *p*-value < 0.005 were selected as potential biomarkers associated with breast cancer patients using the TCGA dataset. As shown in Figure 6, survival analysis of highly ranked candidates (hsa-miR-122, hsa-miR-138, hsa-miR-150, and hsa-miR-204) showed them to be associated with the overall survival of breast cancer patients, by comparing the difference between the high-risk and low-risk groups. In conclusion, the author demonstrated the prognostic power of differentially expressed miRNAs in breast cancer incidence, as well as the effectiveness of MDMF in discovering potential biomarkers.

### 3.6. Pathway Analysis

**Figure 7:**
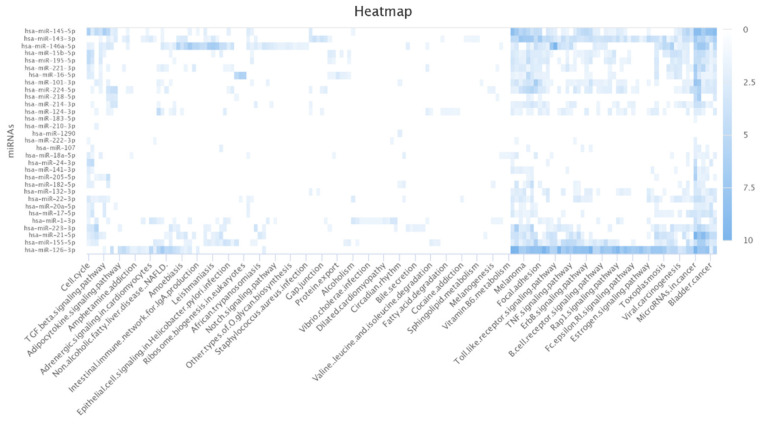
Heatmap of lung cancer-related miRNAs versus target pathways.

The author further conducted the functional analysis of miRNAs based on their targets. DIANA-mirPath v 3.0 is a web-based tool that provides biological pathways related to miRNA targets of interest [48]. I found that most of the targets of the lung cancer-related miRNAs identified by MDMF were associated with lung cancer-related biological processes and functions. For example, non-small cell lung cancer (NSCLC) is the most lethal factor in lung cancer-related mortality (Table 7) [49]. Furthermore, studies have revealed a link between HIF-1 protein and apoptosis and proliferation in lung cancer [50]. Figure 7 shows a heatmap illustrating the relationship between miRNA targets and their target pathways, generated using the miRpathDB 2.0 tool [51]. A darker color represents more relevance in the corresponding pathway functions. In summary, Gene Ontology (GO) analysis and KEGG pathway analyses clearly demonstrated the robustness and effectiveness of MDMF in detecting disease-related miRNAs.

## 4. Conclusions and Future Perspective

Researchers are now focusing on the vital role of miRNAs in disease incidence to better understand complex human diseases. Numerous computational models have been developed to identify disease-related miRNAs by reducing the cost and time for clinical experiments. Aiming at modeling miRNA–disease association prediction model more accurately, the author proposes a novel computational framework, called MDMF, which naturally fuses the Gaussian interaction profile kernel similarity, disease semantic similarity, and miRNA expression values into a matrix factorization model. The matrix factorization-based model developed in this study combines different biological datasets. The high performance of MDMF is attributed to several factors. First, I measured comprehensive disease similarities through the incorporation of heterogeneous data. Second, based on the intuition that incorporating additional information leads to enhanced prediction accuracy, I integrated the Gaussian interaction profile kernel similarity, disease semantic similarity, and miRNA expression values, by adjusting the matrix factorization objective function. Finally, I used the recommender algorithm, matrix factorization, which maps miRNAs and diseases into a shared latent space to predict disease-related miRNAs, resulting in satisfactory prediction accuracy. However, there is still room for improvement in prediction accuracy. Due to the nature of matrix factorization, there is a limitation of capturing only linear relationships by taking inner product of each latent space. Therefore, applying various machine-learning models that can capture non-linear relationships will further improve the prediction performance. From this perspective, the authors plan to develop a hybrid model that combines a linear model and a non-linear model in a future study. The performance of MDMF could be further improved if more biological data are available for the model, such as data regarding target genes and environmental factors. Furthermore, extracting accurate miRNA and disease features using more sophisticated machine learning models can also improve performance.

## Figures and Tables

**Table 1:** Notations.

Symbol	Description
*p*, *q*, *k*	Number of miRNAs, diseases and latent dimensions
*M*, *D*	miRNA and disease latent matrix
*S*	Disease similarity matrix
*W*	miRNA similarity matrix
*L*	Objective function
*λ*	Hyper parameter for regularization
*η*	Learning rate

**Table 2:** Performance comparison of MDMF with other methods (global LOOCV). Various experiments under the various evaluation metrics clearly demonstrated the comparable performance of MDMF. Local leave-one-out cross-validation (LOOCV); Area under the ROC curve (AUCs); Area under the precision-recall curve (AUPR); F1-measure (F1); accuracy (ACC), Matthews correlation coefficient (MCC).

Methods	AUC	AUPR	F1	ACC	MCC
MDMF	0.9147	0.8408	0.8482	0.8547	71.54
MDHGI	0.9040	0.8404	0.8745	0.8391	64.49
PMAMCA	0.8967	0.8501	0.8802	0.8446	68.71
MCMDA	0.8768	0.8043	0.8704	0.8342	63.48
RLSMDA	0.8588	0.7647	0.7342	0.8164	65.42
RKNNMDA	0.7750	0.8482	0.8703	0.8128	64.81

**Table 3:** Performance comparison of MDMF with other methods (local LOOCV). Various experiments under the various evaluation metrics clearly demonstrated the comparable performance of MDMF.

Methods	AUC	AUPR	F1	ACC	MCC
MDMF	0.8905	0.8129	0.8347	0.8538	69.84
PMAMCA	0.8693	0.8846	0.8284	0.8404	62.49
MDHGI	0.8427	0.8104	0.8591	0.8349	66.91
RKNNMDA	0.8292	0.8864	0.8028	0.8116	64.82
RWRMDA	0.7937	0.7372	0.7729	0.7527	59.42
MCMDA	0.7850	0.8764	0.8418	0.8268	68.16
RLSMDA	0.7463	0.8648	0.8045	0.8143	62.72

**Table 4:** The effects of disease constraint on the performance of MDMF.

α	AUC (Global LOOCV)	AUC (Local LOOCV)
0.1	0.8874	0.8711
0.2	0.8954	0.8724
0.3	0.9018	0.8769
0.4	0.9082	0.8784
0.5	0.9127	0.8891
0.6	0.9116	0.8842
0.7	0.9147	0.8905
0.8	0.9128	0.8874
0.9	0.9042	0.8842

**Table 5:** Top 50 breast cancer-related miRNAs predicted by MDMF and their evidence. All miRNA candidates were proved to be related with breast cancer.

Rank	Name	Evidence	Rank	Name	Evidence
1	hsa-miR-214	hmdd, dbDEMC	26	hsa-miR-1237	dbDEMC
2	hsa-miR-937-3p	dbDEMC	27	hsa-miR-129	hmdd, dbDEMC
3	hsa-miR-1248	hmdd, dbDEMC	28	hsa-miR-340*	dbDEMC
4	hsa-miR-920	dbDEMC	29	hsa-miR-16-1-3p	dbDEMC
5	hsa-miR-520e	dbDEMC	30	hsa-miR-302b*	dbDEMC
6	hsa-miR-593	dbDEMC	31	hsa-miR-1266	hmdd, dbDEMC
7	hsa-miR-381	hmdd, dbDEMC	32	hsa-miR-1249-3p	dbDEMC
8	hsa-miR-16	hmdd, dbDEMC	33	hsa-miR-1262	dbDEMC
9	hsa-miR-502	hmdd	34	hsa-miR-494-3p	dbDEMC
10	hsa-let-7g*	dbDEMC	35	hsa-miR-1911*	dbDEMC
11	hsa-miR-370	hmdd, dbDEMC	36	hsa-miR-376b-3p	dbDEMC
12	hsa-miR-330	dbDEMC	37	hsa-miR-1276	dbDEMC
13	hsa-miR-452	hmdd, dbDEMC	38	hsa-miR-331-5p	dbDEMC
14	hsa-miR-124a-3	hmdd, miR2disease	39	hsa-miR-302e	dbDEMC
15	hsa-miR-410-3p	dbDEMC	40	hsa-miR-361-5p	dbDEMC
16	hsa-miR-500a	dbDEMC	41	hsa-miR-205	hmdd, miR2disease, dbDEMC
17	hsa-miR-766-3p	dbDEMC	42	hsa-miR-215-5p	dbDEMC
18	hsa-miR-29a-3p	dbDEMC	43	hsa-miR-30b-3p	dbDEMC
19	hsa-miR-23a	hmdd, dbDEMC	44	hsa-miR-760	hmdd, dbDEMC
20	hsa-miR-3653-3p	dbDEMC	45	hsa-miR-4458	dbDEMC
21	hsa-miR-513b	dbDEMC	46	hsa-miR-30c	hmdd, dbDEMC
22	hsa-miR-125a-3p	dbDEMC	47	hsa-miR-3121-5p	dbDEMC
23	hsa-let-7a-2-3p	dbDEMC	48	hsa-miR-609	dbDEMC
24	hsa-miR-3130-2	hmdd	49	hsa-miR-21*	dbDEMC
25	hsa-miR-1272	dbDEMC	50	hsa-miR-7705	dbDEMC

**Table 6:** Top 50 lung cancer-related miRNAs predicted by MDMF and their evidence. All miRNA candidates were proved to be related with lung cancer.

Rank	Name	Evidence	Rank	Name	Evidence
1	hsa-miR-214	hmdd, dbDEMC	26	hsa-miR-1237	dbDEMC
2	hsa-miR-937-3p	dbDEMC	27	hsa-miR-129	hmdd, dbDEMC
3	hsa-miR-1248	hmdd, dbDEMC	28	hsa-miR-340*	dbDEMC
4	hsa-miR-920	dbDEMC	29	hsa-miR-16-1-3p	dbDEMC
5	hsa-miR-520e	dbDEMC	30	hsa-miR-302b*	dbDEMC
6	hsa-miR-593	dbDEMC	31	hsa-miR-1266	hmdd, dbDEMC
7	hsa-miR-381	hmdd, dbDEMC	32	hsa-miR-1249-3p	dbDEMC
8	hsa-miR-16	hmdd, dbDEMC	33	hsa-miR-1262	dbDEMC
9	hsa-miR-502	hmdd	34	hsa-miR-494-3p	dbDEMC
10	hsa-let-7g*	dbDEMC	35	hsa-miR-1911*	dbDEMC
11	hsa-miR-370	hmdd, dbDEMC	36	hsa-miR-376b-3p	dbDEMC
12	hsa-miR-330	dbDEMC	37	hsa-miR-1276	dbDEMC
13	hsa-miR-452	hmdd, dbDEMC	38	hsa-miR-331-5p	dbDEMC
14	hsa-miR-124a-3	hmdd, miR2disease	39	hsa-miR-302e	dbDEMC
15	hsa-miR-410-3p	dbDEMC	40	hsa-miR-361-5p	dbDEMC
16	hsa-miR-500a	dbDEMC	41	hsa-miR-205	hmdd, miR2disease, dbDEMC
17	hsa-miR-766-3p	dbDEMC	42	hsa-miR-215-5p	dbDEMC
18	hsa-miR-29a-3p	dbDEMC	43	hsa-miR-30b-3p	dbDEMC
19	hsa-miR-23a	hmdd, dbDEMC	44	hsa-miR-760	hmdd, dbDEMC
20	hsa-miR-3653-3p	dbDEMC	45	hsa-miR-4458	dbDEMC
21	hsa-miR-513b	dbDEMC	46	hsa-miR-30c	hmdd, dbDEMC
22	hsa-miR-125a-3p	dbDEMC	47	hsa-miR-3121-5p	dbDEMC
23	hsa-let-7a-2-3p	dbDEMC	48	hsa-miR-609	dbDEMC
24	hsa-miR-3130-2	hmdd	49	hsa-miR-21*	dbDEMC
25	hsa-miR-1272	dbDEMC	50	hsa-miR-7705	dbDEMC

**Table 7:** The effects of disease constraint on the performance of MDMF.

KEGG Pathway	*p*-Value
Hippo signaling pathway	1.41440646708 × 10^−7^
Chronic myeloid leukemia	6.87396730677 × 10^−6^
TGF-beta signaling pathway	7.52715819175 × 10^−6^
ECM-receptor interaction	1.33810742874 × 10^−5^
FoxO signaling pathway	7.94489535244 × 10^−5^
Prostate cancer	0.00245651291245
Non-small cell lung cancer (NSCLC)	0.00329923289869
Thyroid cancer	0.00715240823084
ErbB signaling pathway	0.00817122414933
Pancreatic cancer	0.0120595309627
p53 signaling pathway	0.022215235485
HIF-1 signaling pathway	0.0429548116057

## Data Availability

Not applicable.
